# Interpreting Intervention Induced Neuroplasticity with fMRI: The Case for Multimodal Imaging Strategies

**DOI:** 10.1155/2016/2643491

**Published:** 2015-12-29

**Authors:** Lee B. Reid, Roslyn N. Boyd, Ross Cunnington, Stephen E. Rose

**Affiliations:** ^1^The Australian e-Health Research Centre, CSIRO, Brisbane, QLD 4029, Australia; ^2^Queensland Cerebral Palsy and Rehabilitation Research Centre, School of Medicine, The University of Queensland, Brisbane, QLD, Australia; ^3^School of Psychology and Queensland Brain Institute, The University of Queensland, St. Lucia, Brisbane, QLD, Australia

## Abstract

Direct measurement of recovery from brain injury is an important goal in neurorehabilitation, and requires reliable, objective, and interpretable measures of changes in brain function, referred to generally as “neuroplasticity.” One popular imaging modality for measuring neuroplasticity is task-based functional magnetic resonance imaging (t-fMRI). In the field of neurorehabilitation, however, assessing neuroplasticity using t-fMRI presents a significant challenge. This commentary reviews t-fMRI changes commonly reported in patients with cerebral palsy or acquired brain injuries, with a focus on studies of motor rehabilitation, and discusses complexities surrounding their interpretations. Specifically, we discuss the difficulties in interpreting t-fMRI changes in terms of their underlying causes, that is, differentiating whether they reflect genuine reorganisation, neurological restoration, compensation, use of preexisting redundancies, changes in strategy, or maladaptive processes. Furthermore, we discuss the impact of heterogeneous disease states and essential t-fMRI processing steps on the interpretability of activation patterns. To better understand therapy-induced neuroplastic changes, we suggest that researchers utilising t-fMRI consider concurrently acquiring information from an additional modality, to quantify, for example, haemodynamic differences or microstructural changes. We outline a variety of such supplementary measures for investigating brain reorganisation and discuss situations in which they may prove beneficial to the interpretation of t-fMRI data.

## 1. Introduction

Broadly speaking, “neuroplasticity” refers to the phenomenon of neurons and neural networks modifying their connections and/or behaviour in response to new information, sensory stimulation, development, damage, or dysfunction. The ultimate goal of neurorehabilitation is to induce neural plasticity in a manner that restores the full original function and potential of the injured brain (“neurological restoration”), but a variety of other patterns of neural plasticity may also occur during recovery, including compensatory activity, use of redundant networks, or changes in behavioural or cognitive strategy. Direct measures of such changes are critical to understanding how and when recovery from brain injury takes place and ultimately may lead to improved or novel rehabilitative treatments. One very popular modality used to measure neuroplasticity is task-based functional MRI (t-fMRI). This technique infers from local changes in cerebral blood flow (CBF) to identify brain regions that are more “active” while subjects execute a task than during a comparison or resting state. For a more in-depth explanation of fMRI, readers are referred to Logothetis [1].

The accessibility and noninvasive nature of fMRI are important strengths. When used to measure neuroplasticity, however, t-fMRI suffers from a unique set of challenges that are not always fully acknowledged. With the accelerating development of neurorehabilitation strategies researchers need to be cognisant of the limitations of commonly used neuroimaging technologies, including t-fMRI, in order to collect information capable of advancing our understanding of the neurorehabilitative process. In particular, it is critical that researchers can correctly interpret what a change in t-fMRI signal reflects, if they are to understand the mechanisms of functional recovery.

To aid researchers in this regard, this review explores two important questions: “What are the challenges in interpreting changes in t-fMRI signal as intervention-induced neuroplasticity?” and “How can complementary information from other modalities aid such interpretations?” To contextualise our discussion, we define four basic criteria that we believe are essential for informative interpretation of any neuroimaging signal change in terms of brain changes. We propose that detected changes should (1) be moderately stable or evolve reliably, (2) be meaningfully distinguishable from day-to-day variation in brain activity, (3) offer biological insight into the recovery process, and (4) reliably relate to (or influence) clinical changes. These criteria are somewhat straightforward: to advance neurorehabilitative science, reported changes must be unambiguous, reliable, related to recovery, and clearly a direct or indirect effect of the intervention at hand.

With this in mind, we begin this review by outlining t-fMRI findings associated with intervention-induced neuroplasticity and discuss uncertainties surrounding their interpretations. We highlight that change in t-fMRI activation patterns can be difficult to extrapolate to brain reorganisation and, in some cases, may be confounded by processing inherent to the technique. We follow this overview by offering supporting strategies, focussing on the supplementation of t-fMRI findings with information from other modalities, such as structural MRI or transcranial magnetic stimulation (TMS). Examples are provided as to how incorporating such information can improve interpretation of t-fMRI data, strengthening specific claims about intervention driven neuroplasticity.

Though some points made here may be generalised to other contexts, this commentary restricts discussion to studies targeting motor impairment and movement rehabilitation in patients with cerebral palsy (CP) or acquired brain injuries, such as traumatic brain injury (TBI). As relevant literature describing therapy-driven brain reorganisation is limited in patients with acquired brain injuries, we also make reference to neuroimaging studies based on adult stroke populations and some nonlongitudinal studies. It must be kept in mind that while subject groups may all undergo neuroplasticity in response to rehabilitation, they may do so from a vastly different baseline, particularly due to the impacts of brain injury on early development [2]. Further, for the sake of brevity, discussion here is restricted to standard GLM-analyses of t-fMRI, as this is the dominant technique in published literature; resting state fMRI and other forms of fMRI are not considered.

## 2. Common Findings

**Figure 1:**
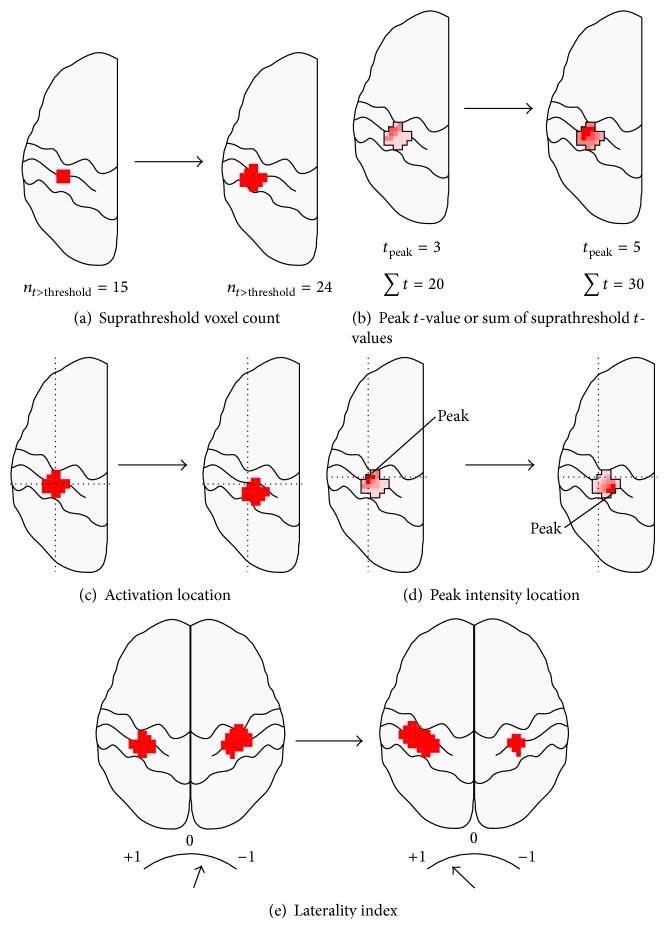
Commonly seen fMRI activation changes. Common fMRI activation pattern changes reported in the literature include suprathreshold voxel counts (a), peak *t*-values or sum of suprathreshold *t*-values (b), activation location (c), peak *t*-value location (d), and changes in laterality index (e). Some studies report changes over time, while others report differences between groups. The degrees of changes shown here are for illustrative reasons only.

There are three primary findings that are commonly reported in t-fMRI studies of neurorehabilitation, summarised in Figure 1.

### 2.1. Intensity and Size Changes

**Figure 2:**
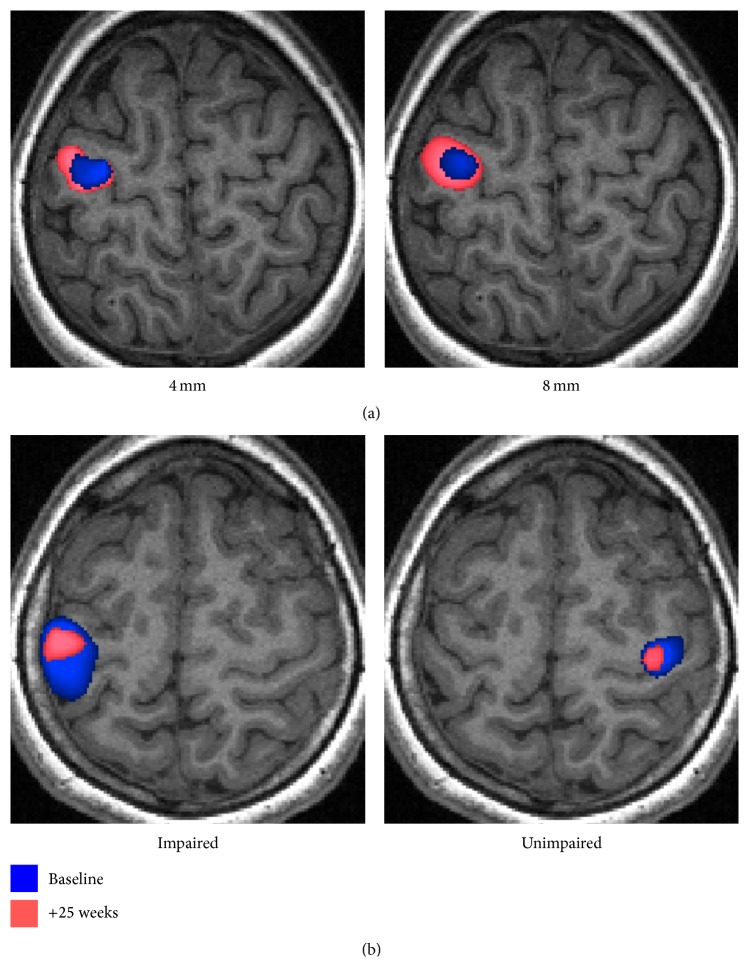
Voxel-wise fMRI analyses of a block-design hand-tapping task recorded at baseline (blue) and after ~25 weeks (pink). (a) A subject with chronic traumatic brain injury who underwent virtual reality therapy during the 25-week period. In the affected hemisphere, the 25-week scan showed a 2.2 times or 3.3 times larger activation volume than the baseline scan, depending on whether a 4 mm (left) or 8 mm (right) smoothing kernel was used. The 4 mm and 8 mm processing options were associated with peak voxel shifts of 8.6 mm and 5 mm, respectively. (b) A subject with cerebral palsy demonstrating large changes in activation between scans, for tapping of the impaired (left image) and unimpaired (right image) hands. The subject underwent no treatment during the 25-week period but was less anxious and followed the auditory cue marginally more accurately during the follow-up scans.

Altered ipsilesional activated-voxel counts, or heightened peak intensities, are commonly reported for patients with brain injuries who have received treatment, improved function, or when compared with controls (Figures 1(a), 1(b), and 2(a)).

Heightened activation of motor regions has been reported for children with TBI [11] and adolescents with CP [12] when compared with controls. A recent systematic review reported seven longitudinal t-fMRI studies of treatment interventions for unilateral CP, drawn from four unique subject cohorts [13]. After therapy, area of activation of the (most) impaired hemisphere reportedly increased in a subset of subjects within each study [13].

In TBI, one study of seven adult subjects with primarily-nonchronic injury showed changes in the activation volumes of several sensorimotor-related regions in response to motor rehabilitation [14]. The location and relative changes in activation volumes varied greatly between subjects. Increased ipsilesional premotor activation has been shown in response to constraint-induced movement therapy, alongside improvements in Fugl Meyer assessment scores, in a single adult with chronic traumatic damage to the primary sensorimotor cortex (S1M1) [15]. Similarly, increased S1M1 activation has been found in two adult TBI subjects after robotic therapy [16]. Likewise, following adult stroke, regions of sensorimotor activation are reportedly larger in recovered patients than in partially recovered patients [17] and can further enlarge with motor training [18].

### 2.2. Laterality Shifts

The second common t-fMRI finding in patients with brain injuries is a shift in the hemispheric-balance of activation (Figure 1(e)). In normal subjects, basic motor tasks overwhelmingly activate the contralateral S1M1 [19]. Both stroke and unilateral CP patients, however, regularly demonstrate robustly bilateral activation [12, 20, 21]. These balances of activation are typically calculated as laterality index (LI):

(1) $$\begin{array}{c} \text{LI}=\frac{\left(\sum C-\sum I\right)}{\left(\sum C+\sum I\right)}, \end{array}$$

where ∑*C* and ∑*I* are suprathreshold voxel counts or *t*-value sums (weighted LI, also referred to here as LI for simplicity), for the contralateral and ipsilateral hemispheres, respectively. LIs fall between −1 (only ipsilateral activation) and +1 (only contralateral activation).

In stroke, S1M1 LI values for the paretic hand are lowest in acute stroke, due to both decreased ipsilesional activity and increased contralesional activity [21]. Over time, these values become more positive [18, 21] but do not typically return completely to “normal” values [22], even in well-recovered patients [17, 23]. In chronic stroke patients, LI values are often [24, 25], but not always [26], reported to shift toward the lesioned hemisphere in response to rehabilitative therapy.

In children with unilateral CP, activation of ipsilateral sensorimotor regions can be evoked with active movements, passive movements, and tactile stimulation of the impaired limb [12], the patterns of which depend on their type of reorganisation [6, 27]. Small-scale studies of children with unilateral CP suggest that virtual reality and constraint-induced movement therapies can alter the balance of activation toward the contralateral hemisphere [13, 28]. This may prove functionally beneficial: contralateral somatosensory activation during motor tasks appears to be associated with improved unimanual capacity [19].

Numerous studies have proposed that laterality shifts demonstrate an adaptive bihemispheric reorganisation of motor networks [22, 23, 25, 28, 29]. This is a key point that we will return to later.

### 2.3. Intrahemispheric Relocation of Activation

Differences in intrahemispheric location of S1M1 activation, between either time points or subject groups, are also frequently reported as evidence of neurological reorganisation (Figures 1(c) and 1(d)). This metric is principally reported in adult stroke literature, where longitudinal dorsal “shifts” in peak activation have been described 4, 12 [18], and 24 months after stroke [30]. Different loci of activation have been reported between stroke and control subjects numerous times [17, 31]. One study [32] has reported a correlation between peak S1M1 activity location and motor impairment.

## 3. The Challenge

It is clear that changes in t-fMRI measures have been reported in a variety of studies and pathologies. This section identifies several challenges that make the interpretation of such results in terms of neuroplasticity particularly difficult. These issues include subject variability, biological ambiguity, methodological considerations, and confounds introduced by disease states. As we shall discuss, these factors impede informative interpretation of the t-fMRI signal by obscuring two key facts: (1) whether neurological change has genuinely taken place and (2) if so, what type of change has been observed. Possible solutions to reduce the impact of these variables are summarised within the final section of this review. These incorporate the use of information from other modalities within the study design, providing complementary support for t-fMRI measured brain changes, to provide more robust evidence of neuroplasticity.

### 3.1. t-fMRI Results Are Variable

One of the greatest challenges for t-fMRI in studies of neurorehabilitation is the heterogeneity in findings, both within and between studies of patients with brain injury. Intrahemispheric “relocations” of activation, for example, are not always reported and have been variable even within studies, differing, for example, by patient subgroup [17] or task performed [30]. In addition, changes in activation patterns do not consistently correlate with behavioural improvements (Figure 2(b)). Distinct changes in activation patterns have been reported in rehabilitative studies of adult stroke (postrehabilitation versus retention) [18], hemispherectomy (pre- versus postrehabilitation) [33], and paediatric CP (pre- versus postrehabilitation) [34], despite subjects demonstrating stable motor scores. In unilateral CP, the degree of S1M1 activation for active and passive movements may not correlate with motor scores [12, 20], and results for sensory impairment are mixed [8, 12, 35]. Similarly, for stroke, activation of the ipsilateral primary motor cortex has been associated with both good and poor behavioural outcomes [21]. Such variability can render the physiological significance of t-fMRI differences unclear.

One probable source of this heterogeneity is patient variability. Factors such as anatomical location, extent, type, and timing of insult can have profound influences on neurological impairments, response to treatment, and the type of neuroplasticity required for recovery [36]. Controlling for such factors can be very difficult. Restricting a study to patients in the chronic stage of injury, for example, may not remove effects due to progressive Wallerian degeneration and/or volumetric changes, which take place during the first few years following stroke [37] and, potentially, TBI [38]. Response to treatment also appears to be subject to intact contralateral corticothalamic connections in stroke subjects [39] and ipsilateral corticospinal connections in children with CP [27]. Such factors can dramatically alter the interpretation and biological significance of measures such as LI, but their identification requires utilisation of additional modalities, such as TMS or diffusion imaging.

Attempts to limit such variability is probably one reason why most t-fMRI studies investigating neuroplasticity include only ~4–10 subjects with brain injury [13, 21]. Reproducibility studies have demonstrated that even well-controlled longitudinal t-fMRI studies of normal subjects likely have a high degree of intrasubject measurement error [40] and require at least 20 subjects per group to perform reliable and sensitive group analyses [41]. The higher degree of variability seen within brain injury cohorts means that required numbers are likely to be substantially higher.

### 3.2. Biological Ambiguity

It is common in the t-fMRI literature to refer to activation differences as direct evidence of adaptive neuroplasticity. What is rarely addressed is the fact that activation differences, in isolation, do not allow researchers to differentiate between a variety of substantially different biological processes, many of which do not indicate regained, novel, or improved neurological capabilities and may not be positive or adaptive at all.

#### 3.2.1. Compensatory Activation

One of the most obvious alternative explanations to adaptive neuroplasticity is that activation changes reflect normal system dynamics* compensating* for poor performance. One possible compensatory method is more intensive processing in already-activated tissue. This ties in with the topic of task equivalency and is discussed later. Another mechanism is the compensatory activation of redundant motor areas [42].

It is already established that the brain can switch between apparently functionally equivalent sensorimotor representations in response to disrupted activity, for example, during a tumour removal operation [43], or reversibly within minutes of direct inactivation of motor areas [44]. Equivalent dynamics are probably, then, likely to occur in brain injury. Importantly, t-fMRI alone is unable to determine whether such dynamics reflect a switch (1) to an equipotent area (reflecting ongoing impairment), (2) back to the original area (restored function), or (3) to an area previously incapable of such responsibility (novel gain in function). Given the three distinctively different take-home messages for the intervention investigated, there is a strong argument for researchers to seek secondary evidence (e.g., microstructural, conduction, or connectivity changes) before assuming that an activation change necessarily indicates novel or regained function.

#### 3.2.2. Strategic Shifts

Rather than relying on neurological recovery, subjects can improve task performance by altering the role of muscle groups, improved motor planning, or better attending to feedback. Some adult stroke patients rely more heavily on proprioceptive feedback than healthy subjects [45], for example. Adult stroke patients have also been shown to adopt compensatory movement patterns, including atypical-muscle use for pointing and reaching tasks, during rehabilitation [46, 47]. Importantly, such compensation can result in “improved” motor scores, despite unimproved motor capabilities [46], and is associated with poorer recovery [47]. In addition, studies combining TMS and t-fMRI have revealed that attention, anticipation, and/or the forward-planning of motor movements dramatically alters cortical excitability in button pressing tasks [48, 49].

Given these points, it is not unreasonable to surmise that subtly different behavioural strategies may underlie subtle changes in t-fMRI activation patterns. While it could be argued that learning is a form, or the result, of neuroplasticity, again the usability of information becomes limited if one cannot differentiate between “working around” ongoing disability and neurological restoration.

#### 3.2.3. Task Difficulty

Task equivalency is another, related, source of uncertainty in t-fMRI. Typically, studies have all subjects perform identical tasks at all time points. One argument is that controlling for differential performance is essential to avoid different workloads or feedback confounding results (Figure 2(b)) [50]. In order to perform similarly to controls, however, impaired patients have to apply more effort or execute different strategies, such as a more heavy reliance on feedback, which can increase recruitment of S1M1, attentional networks, and/or supplementary areas [11]. These sustained attentional demands are also more difficult for brain injured subjects to meet [51–53] and may influence activation of some sensorimotor areas, independently of motor output [48]. Increased cognitive fatigue may also result in more frequent head movement, which can impact analyses [54]. To avoid this issue, the equivalency of perceived effort can, instead, be controlled for (e.g., by modulating the range of motion or force exerted). Subjects performing different tasks, however, may use different task strategies, musculature, and/or receive different somatosensory feedback, all of which may alter activation patterns. In some instances, it may be possible to conduct two tasks, one controlling for perceived effort and another where performance is controlled between participants. These two sets of functional results can then be interpreted in the context of one another and the behavioural observations noted during scanning. Researchers should carefully consider their participants before selecting this course of action as the attention required to perform multiple tasks without head movement may be beyond the means of young children, people with moderate-to-severe disability, and participants with acute brain injury (such as concussion).

Another option is the use of trivial tasks with limited cognitive load, for which perceived task difficulty and performance are likely to be identical across sessions. Scans using these tasks, however, may be insensitive to subtle reorganisation and would require exclusion of most moderately impaired patients, for whom no task is “trivial.” Passive movements of the impaired limb are a final option [19] but may miss genuine activity and reorganisation associated with motor planning and execution [12]. As such, most rehabilitation studies that incorporate fMRI of motor tasks are best positioned by accepting the task equivalency problem, choosing a simple/stable task, and making claims in the context of secondary, independent evidence of neurological change.

#### 3.2.4. Disinhibition

Shifts in LI toward the contralesional hemisphere have been previously interpreted as neuroplastic compensation for a damaged sensorimotor cortex. At least in stroke, however, contralesional activation does not appear to be a good predictor of functional recovery [55].

**Figure 3:**
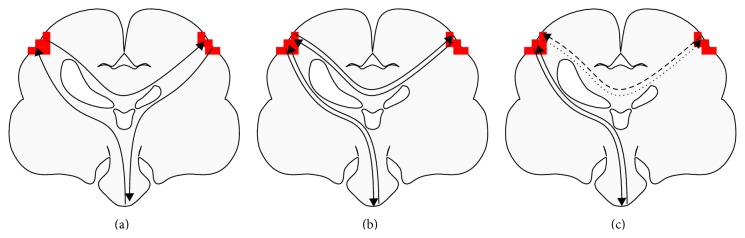
Task-based fMRI activation changes, in the presence of bilateral activation, require additional information for useful interpretation. Activation changes are often interpreted with the assumption that sensorimotor processing occurs primarily contralaterally, with interhemispheric relaying of information for supplementary processing (b). In cerebral palsy, however, sensory processing is often contralaterally organised, while motor signals emanate from the ipsilateral hemisphere (a). In addition, in cerebral palsy, stroke, and acquired brain injuries, imbalances in interhemispheric inhibition ((c); dashed lines) may be the primary factor influencing t-fMRI activation. Such organisations, and thus meaning of t-fMRI activation changes, can be elucidated via supplementary methods such as TMS and MEG recordings.

Given that the motor cortices inhibit one another in normal subjects [56], an alternative explanation is interhemispheric disinhibition (Figure 3): damage to the lesioned hemisphere reduces its inhibitory ability, leading to contralesional hyperactivation. TMS and fMRI + TMS studies have provided direct evidence for this hypothesis in subjects with CP [57], TBI [58], and stroke [17, 59]. Contralesional activity may even have a net-negative influence: direct inhibition of such activity with transcranial direct current stimulation can improve motor scores [60] and motor-skill acquisition [61] in adults with chronic stroke.

Unknown anatomy and functional dynamics can further undermine interpretation of changes in LI. In CP, preserved ipsilateral corticospinal connections may exist [6], which t-fMRI-only studies are unable to discern. In stroke, one fMRI + TMS study revealed that contralesional dorsal-premotor-cortex activity was correlated with poorer clinical scores, facilitating the ipsilesional motor cortex in impaired patients but inhibiting it in patients exhibiting good recovery [32]. These results highlight how difficult correctly interpreting t-fMRI activation differences can be in subjects with impairment. Activations may be adaptive, maladaptive, pathological, excitatory, inhibitory, and/or net-neutral. Which interpretation is correct is something that cannot be determined by t-fMRI alone.

### 3.3. Methodological Considerations

#### 3.3.1. Smoothing

There are methodological considerations to consider when evaluating changes, or differences, in the spatial extent and location of t-fMRI activation peaks. Smoothing of voxel intensities is a common step in t-fMRI analyses and varies greatly between studies, often without supplied justification [62]. Smoothing can have dramatic nonlinear effects on voxel variances which can alter the volume and shape of activation, as well as the location of peaks (Figure 2(a)) [63]. Even kernels as small as 4 mm can shift peak-intensity localisation of motor centres by several millimetres [64]. Such effects should be kept in mind when inferring from activation characteristics, especially with larger smoothing kernels, which are more optimal for the small cohort sizes seen in this field.

#### 3.3.2. Spatial Normalisation

When conducting group analyses, it is typical to nonlinearly register scans to a standardised “normal brain” template. This normalisation step can, however, inappropriately distort the location of tissues surrounding brain lesions [65]. This may lead to shifts in activation location and activation-size differences between groups in damaged hemispheres. Performing affine-only registration, cost-function marking, or unified segmentation may reduce such effects but does not guarantee their elimination [66]. These effects should be given consideration when interpreting group-wise analyses, especially given that reported location differences are typically in the millimetre range and derived from small sample sizes.

#### 3.3.3. Cluster Analyses

Care must also be taken with interpretation of cluster analyses, which comprise the majority of recent t-fMRI analyses [62, 67]. A cluster of voxels discovered through a cluster analysis does not infer that all voxels within that cluster were significantly active during the task. A cluster indicates a region that meets a minimum size requirement, somewhere inside of which there is evidence against the null hypothesis [68–70]. A consequence of this is that the spatial specificity of these analyses is typically low, especially with larger clusters [70], and one cannot make specific inferences about particular voxels within the cluster. When studying neuroplasticity, an enlarged cluster does not, thus, necessarily mean that neurons on the periphery of that region are newly utilised for a task. Similarly, a cluster that has changed shape or shifted slightly may still only have the “true” activation in the same location. This is of particular concern when liberal primary voxel-level thresholds (e.g., *p* < 0.01) are used, as these further dilute the ability to make claims about spatial location of activation [70]. Use of liberal thresholds is not uncommon: a recent review of 814 cluster-based fMRI studies published in high-impact journals described use of liberal thresholds as “both endemic and detrimental to the neuroimaging field” [67].

## 4. Disease Confounds

Beyond their most obvious motor impairment, subjects with brain injuries may also present with a number of complicating factors that cannot easily be controlled for between groups or time points and may impact t-fMRI analyses in unexpected ways.

### 4.1. Acute Effects

In acute and subacute stages of brain injury, fMRI signal may be heavily influenced by temporary vascular changes [71]. Evolution of activity patterns during this time may also simply demonstrate the temporary effects of a regressing oedema, mass effect, and/or inflammation, all of which are expected to acutely impact function [3]. As such, special care should be taken not to misconstrue t-fMRI changes during early disease states as neuroplasticity, without secondary evidence ruling out such causes.

### 4.2. The Haemodynamic Response Function

Standard BOLD analyses rely on a number of assumptions, including that neurovascular coupling (1) is consistently overcompensatory, (2) is adequately regionally invariant, and (3) has a sufficiently standard time-course between regions and subjects. These assumptions may be invalidated by the substantial cerebrovascular damage that is associated with many forms of stroke, TBI, and congenital hemiplegia. Altered CBF has been reported for all clinical stages of both stroke [72–74] and TBI [71, 75]. Stroke patients' haemodynamics may be additionally impacted nonglobally by concurrent vascular disease caused by risk factors such as advanced age, smoking, hypertension, and diabetes mellitus.

As normal haemodynamic responses overcompensate for metabolic needs, reduced cerebrovascular reactivity can present as a diminished BOLD signal, despite unaltered levels of neural activity. As such, in longitudinal designs involving nonchronic patients, it may be impossible to differentiate between changes in neural activation and cerebrovascular reactivity using t-fMRI alone [72]. Of particular concern, several studies have found that the haemodynamic response near a lesioned site is more strongly impacted by injury than nonlesioned regions, even in chronic disease states [71, 73, 74]. Finally, there is evidence that aspects of cerebrovascular reactivity may be correlated with motor performance in certain stroke patients [76], even in the absence of marked vascular disease [77]. It is noteworthy that dynamic causal modelling, a more advanced fMRI analysis method, may be more robust to haemodynamic inhomogeneities by modelling haemodynamics in a region-wise fashion [78, 79].

### 4.3. Head Movement

Head movement can have profound impacts on fMRI signal. Although movement between frames can be reversed through reslicing, there are other sources of signal changes associated with movement (e.g., spin history effects) that will remain. Even after statistical adjustment, submillimetre RMS movement can lead to measurably reduced statistical power [80]. Such movement is more likely in subjects with movement disorders (i.e., dystonia) or reduced cognitive abilities or who find the task difficult [54]. Movement artefacts can be reduced by excluding subjects or censoring frames with movements [80], but this may systematically reduce the statistical power for one subject group and can lead to sampling biases [54].

## 5. Summary and Recommendations

There is little doubt that t-fMRI is an important neuroimaging modality. The aim of this review is not to critique t-fMRI* per se* nor to blanket-prescribe a specific method by which to quantify functional images when measuring neuroplasticity. Rather, we wish to make researchers and clinicians aware of the systematic and methodological challenges affecting common t-fMRI study designs, which are often not addressed or acknowledged, and elucidate how these issues can be mitigated through a multimodal approach. To summarise our case so far, even if confounds such as movement, acute effects, and haemodynamic differences are eliminated, it is still possible that some findings may be explainable by unavoidable data processing steps, such as smoothing and spatial normalisation. These issues are particularly concerning given the vast patient variability and low subject numbers seen in this field. Even when overcoming such issues, assumptions of brain plasticity based on t-fMRI evidence alone are problematic due to difficulties in differentiating between recovery, compensation, use of preexisting redundancies, changes in strategy, and maladaptive processes. In studies of neurorehabilitation, it is critical that researchers can correctly interpret what a change in t-fMRI signal actually* means* in order to understand the mechanisms of functional recovery.

In this review our basic criteria for informative interpretation required that signal changes were moderately stable, meaningfully distinguishable from day-to-day variation, reliably related to clinical changes, and offered biological insight into the recovery process. The first, imperative, step to meeting these criteria with t-fMRI is to relate changes to valid and reliable measures of motor function. Planning longitudinal studies can also provide certainty that any activation changes seen are not due to patient heterogeneity. To overcome the remaining challenges, multimodal imaging can help in four ways. Firstly, multimodal information can allow more homogenous cohorts to be selected or subgroups identified for analysis. Secondly, by providing contextual information, other modalities can narrow down which biological process t-fMRI may have indexed. Relatedly, additional modalities can quantify potentially influential covariates, such as haemodynamic differences, to assess their impact on t-fMRI. Finally, when uncertainties and/or ambiguities are still prevalent, change measured through an independent method can provide confidence that t-fMRI is genuinely indexing a stable functional change. Many multimodal configurations are available that have already proven valuable in helping studies meet these criteria; examples are listed in Table 1.

### 5.1. Structural MRI

Structural MRI allows measurement of cortical thickness: essentially an index of locally or globally available grey-matter. While cortical thickness can be challenging to measure precisely, especially in patient cohorts presenting with cortical lesions or malformations, such analyses are typically automated, simple to visually assess, and can be easily overlaid with t-fMRI statistical parametric maps. Adequate structural images are routinely acquired within fMRI-scan sessions and usually simple to acquire motion-free. While structural imaging is probably less sensitive to change than t-fMRI, these methods share few sources of uncertainty and provide one another with useful contexts for plausible interpretation. In particular, as locally increased grey-matter thickness likely reflects newly ongoing utilisation of that tissue [81], increases in this measure may indicate that any accompanying t-fMRI activation increases are moderately stable and reflect some form of gain-in-function rather than, for example, a switch to an unchanged “backup” network. Changes seen in cortical thickness are particularly beneficial to studies with limited subject numbers, where well-powered group analyses, which can rule out day-to-day variability in neural or vascular dynamics, are difficult or impossible to perform.

Analyses of structural images and diffusion MRI (below) can also quantify potential covariates (such as degeneration, regressing oedema, or developmental maturation) that may affect t-fMRI metrics longitudinally and are likely to vary by subject-cohort and time-point.

### 5.2. TMS

TMS is unique in its ability to directly characterise structural-functional connectivity, including intercortical inhibition, corticospinal tract conductivity, and motor thresholds. TMS may prove particularly useful for studies that need to characterise the functional meaning of t-fMRI determined LI changes. TMS has been used in multiple studies to differentiate between subject subgroups, allowing researchers to understand the biological significance of bilateral fMRI activation patterns in CP [6, 8] and reveal correlations between fMRI changes and long term outcomes in stroke [7].

### 5.3. EEG and MEG

Magnetoencephalography (MEG) and electroencephalography (EEG) can improve certainty in t-fMRI changes by providing direct measures of net neuronal activity that are not likely to be impacted by factors such as haemodynamics, or the aforementioned methodological considerations. The very high temporal resolution of these methods can also allow researchers to distinguish between stages of processing, such as motor planning and execution [28]. Concurrent EEG + fMRI is now possible, although caution may be advised in cohorts for whom head movement is an issue, as concurrent artefacts may result in plausible type-I errors [82].

EEG and MEG information can profoundly change the interpretation of changes in t-fMRI metrics, such LI or activation volume, and elucidate whether comparisons between subject groups are valid. For example, MEG has been used in conjunction with fMRI and TMS to demonstrate that, in some subjects with CP, bilateral t-fMRI S1M1 activation reflects contralateral somatosensory processing alongside ipsilateral (reorganised) motor processing [8]. This illustrates clearly how categorisation of such subjects into homogeneous subgroups can be critical for t-fMRI metrics to be appropriately interpreted (Figure 3). The researchers highlighted that, particularly for motor-based t-fMRI, “[d]efinitively disentangling such bilateral activation is… only possible when complementary methods are used, like TMS and MEG” [8].

### 5.4. PET and ASL

Positron emission tomography and arterial spin labelling are neuroimaging methods that can provide measurements of regional CBF, and so reveal whether haemodynamic differences are affecting fMRI measurements. Arterial spin labelling is a contrast-agent-free MRI technique that can be carried out in ~10 minutes, during the same session as an fMRI. PET is advantageous in that it can additionally provide direct measures of glucose metabolism in brain tissue but requires access to PET imaging equipment and associated radiopharmaceutical facilities. Because both of these methods provide quantitative measures of local haemodynamics, they can quantify precisely how fMRI measurements in each region are affected by factors such as angiogenesis or vascular impairments. This may provide certainty in situations involving lesions, suggest adjustment of haemodynamic parameters, provide guidance on study design (i.e., indicate whether a block-design should be chosen over an event-related design), or shed light on otherwise-unclear findings. In one illustrative study of healthy adults, increases in t-fMRI activation volumes were shown in the supplementary motor area and M1 after two weeks of motor training [10]. These volumes subsequently declined to near-baseline values during the following two weeks of training, despite ongoing improvements in motor performance. PET scans showed that regional CBF increased between all time points, revealing that fMRI decreases were probably due to increased blood flow at rest, rather than actual decreases in brain activity during task execution.

### 5.5. Diffusion MRI

Diffusion MRI (dMRI) measures the directional diffusivity of water in tissue and can provide a variety of useful metrics. In subacute head injury or stroke, dMRI can be used to ensure that t-fMRI differences reflect more than inflammation or oedema. “Microstructural integrity” indices, such as fractional anisotropy and mean diffusivity, can provide evidence that t-fMRI changes represent ongoing changes in brain activity outside of the scanner: these metrics correlate with, and are sensitive to, myelination, which increases in response to ongoing electrical activity [83]. Advanced analyses can identify white matter pathways, calculate their intra-axonal volumes, and index the physical “connection strength” between cortical and subcortical regions. These measures correlate with functional measures in CP [84] and may help provide a more complete picture when interpreting changes between balances of activation between brain regions. Another form of dMRI, neurite orientation dispersion and density imaging [85], provides the opportunity to reveal whether shifts or enlargements of t-fMRI activation reflect local network changes in, for example, the cortex or thalamus [86].

Diffusion MRI data are easily acquired in the same session as an fMRI scan, usually in 8–12 minutes. As dMRI is acquired at rest, overt movement is easier to avoid than with t-fMRI and is unlikely to be correlated with factors such as ability. Standard preprocessing methods can also correct or “scrub” moderately (≤10%) motion-corrupted dMRI data without compromising the final result [87].

### 5.6. t-fMRI Fusion

**Figure 4:**
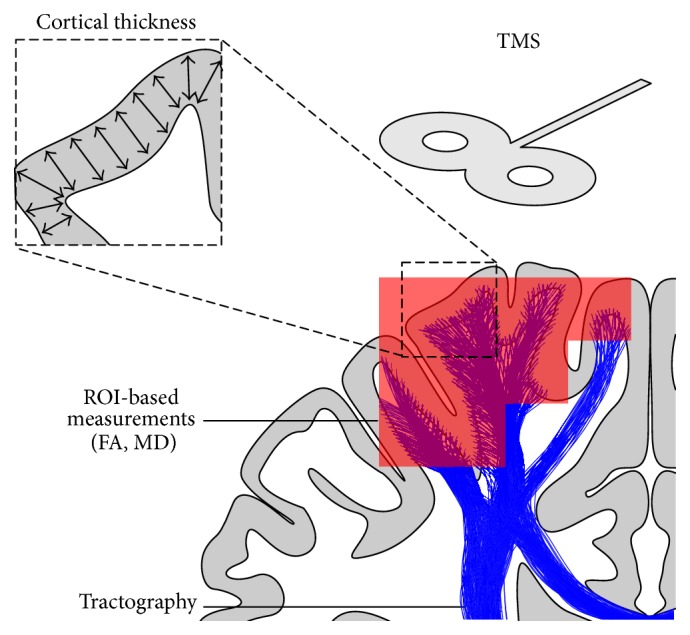
Utilising fMRI in conjunction with a number of other modalities. The red region indicates an area of significant activation, determined with fMRI. When combined with structural MRI, these activated regions can be used as ROIs in which targeted measures of cortical thickness can be made. When combined with diffusion MRI, the fMRI ROIs can be used as sample regions for FA and MD values within subcortical white matter, both of which provide information about tissue microstructure. These ROIs can also act as seed regions to drive tractography, from which white matter connectivity and integrity can be measured. Combining fMRI with TMS measures can also provide context to and certainty about the functional-relevance of fMRI-based findings. ROI: region of interest; FA: fractional anisotropy; MD: mean diffusivity; TMS: transcranial magnetic stimulation.

Finally, a promising alternative approach is to not infer directly from t-fMRI activation patterns, but rather to use t-fMRI to identify functionally important regions-of-interest in which other modalities should make measurements (Figure 4). This fusion of information can avoid some pitfalls of overanalysing changes in activation patterns, while considerably improving the sensitivity and interpretability of other modalities [5, 88]. One fusion method which is being progressively adopted is the use of t-fMRI activation patterns to guide diffusion tractography, allowing this method to focus microstructural and structural-connectivity measurements on functionally relevant areas [4, 5, 88].

### 5.7. Optimising Multimodal Study Designs

Asking a specific research question is fundamental to optimising a study design. Focussed questions (e.g., “How does rehabilitation alter S1M1 connectivity?”) are not only inherently more testable than very broad questions (e.g., “What does rehabilitation change in the brain?”), but can also provide guidance on which study design is appropriately powerful, which modalities and behavioural measures can contribute to the overall picture, and how t-fMRI metrics may require supplementation or disambiguation with additional information. For example, investigations into somatosensory processing may require teasing apart t-fMRI activation using temporally precise signals (MEG) and/or information about integrity of the corticothalamic tracts (TMS or dMRI).

With a specific question in place, one should then consider which factors may primarily impair t-fMRI interpretability. Modalities that can minimise such issues are those which can either quantify their extent or provide supplementary evidence that is unaffected by such issues. For example, if a difference in t-fMRI activation volume is expected between two groups, but one group may have impaired haemodynamics, quantifying regional-CBF with arterial spin labelling, or directly measuring brain activity with MEG, may be of great benefit. As a contrasting example, a t-fMRI study of subjects displaying dyskinesia is unlikely to benefit greatly from dMRI, as both may be confounded by movement artefacts.

Finally, in some situations, the benefits of multimodal imaging may be limited. Studies with very low subject numbers, particularly cross-sectional studies, may see limited benefit from modalities that are less sensitive to change or have high intersubject variance. In such cases, resources may be better spent on boosting subject numbers or collecting additional behavioural information than on additional neuroimaging. In addition, studies that are unable to collect relevant and reliable clinical measures have limited abilities to discern the relevance of neuroplastic changes, regardless of how many imaging measures are taken.

## 6. Conclusion

For measures of neuroplasticity in subjects with brain injuries, the reliability and interpretability of t-fMRI is hampered by a unique set of systematic and methodological challenges. Multimodal imaging provides the opportunity for t-fMRI results to be interpreted with more confidence and biological specificity, ultimately providing greater understanding of the rehabilitative process. Which complementary imaging modality offers the most benefit depends on the study question and subjects selected. Many of these modalities have a minimal time and financial cost for acquisition while still offering exciting, novel opportunities to explore the relationship between structure, function, and clinical outcome which simply cannot be investigated in any other way.

## Figures and Tables

**Table 1:** Example of multimodal studies of brain injury and neuroplasticity.

Reference	Disorder	Additional measures(s)	Significance
Werring et al. [3]	TBI	dMRI	Earliest known combined fMRI + dMRI study for a recovering patient. Combined imaging revealed which corticospinal tracts were partially damaged and whether they were still in use.

Palmer et al. [4]	Healthy subjects	dMRI tractography	fMRI-guided tractography elucidated minute longitudinal structural changes; changes were not detected by fMRI alone.

Cherubini et al. [5]	TBI	dMRI tractography	In patients, fMRI-guided tractography identified additional corticospinal connections and more normal connectivity patterns than atlas-based seeding.

Staudt et al. [6]	CP	dMRI, TMS	TMS, dMRI, and fMRI of motor areas showed good agreement, except in the only successfully scanned subject with bilateral fMRI activation. For this subject, TMS and dMRI ruled out an ipsilateral CST connection.

Rijntjes et al. [7]	Stroke	TMS	Integrity of the pyramidal tract was required for patients to show lasting responses to CIMT. Long term outcomes, fMRI patterns, and correlations between these factors were dependent on such integrity.

Wilke et al. [8]	CP	TMS, MEG	Multimodal imaging demonstrated that sensory organisation was preserved despite motor reorganisation.

Schaechter et al. [9]	Stroke	Cortical thickness	fMRI activations correlated with cortical thickness specifically in putative area 3b of the lesioned hemisphere.

Xiong et al. [10]	Healthy subjects	PET	The fact that fMRI “returns to baseline” in long term motor training may be due to an increased baseline rCBF, rather than the assumed decrease in activation during task performance.

TBI: traumatic brain injury; dMRI: diffusion MRI; CIMT: constraint-induced movement therapy; CP: cerebral palsy; TMS: transcranial magnetic stimulation; MEG: magnetoencephalography; PET: positron emission tomography.
